# High risk of coronary artery obstruction during TAVR, how to avoid it?

**DOI:** 10.1186/s13019-024-02615-z

**Published:** 2024-03-19

**Authors:** Jose R. Gayosso-Ortíz, Juan F. Garcia-Garcia, Jose Alfredo Merino-Rajme, Roberto Muratalla-González, Juan C. Fuentes-Moreno, Arnoldo S. Jiménez-Valverde, Marco A. Alcantara-Melendez, Heberto Aquino-Bruno

**Affiliations:** 1 Interventional cardiology service, National Medical Center, November 20, Mexico City, Mexico; 2 Interventional cardiology service, General Hospital of Mexico, Mexico City, Mexico

**Keywords:** TAVR, Aortic stenosis, Coronary artery obstruction, Chimney stent technique

## Abstract

**Background:**

Coronary artery obstruction after percutaneous aortic replacement is a complication with high short-term mortality secondary to the lack of timely treatment. There are various predictors of coronary obstruction prior to valve placement such as the distance from the ostia, the degree of calcification, the distance from the sinuses; In such a situation some measures must be taken to prevent and treat coronary obstruction.

**Case presentation:**

An 84-year-old male, with severe aortic stenosis and high surgical risk, who was treated with TAVR. However, during the deployment of the valve he presented hemodynamic instability secondary to LMCA obstruction. The intravascular image showed obstruction of the ostium secondary to the displacement of calcium that he was successfully treated with a chimney stent technique.

**Conclusions:**

The high degree of calcification and the left ostium near the annulus are conditions for obstruction of the ostium at the time of valve release; In this context, provisional stenting prior to TAVR in patients at high risk of obstruction should be considered as a safe prevention strategy to achieve the success of the procedure.

**Supplementary Information:**

The online version contains supplementary material available at 10.1186/s13019-024-02615-z.

## Introduction

Although being a less invasive therapeutic modality compared to surgery, the TAVR procedure is associated with potentially serious complications, including acute coronary obstruction.

Coronary artery obstruction (CAO) after percutaneous aortic replacement is a complication with high short-term mortality secondary to the lack of timely treatment [1]. There are various predictors of CAO prior to valve placement such as the distance from the ostia, the degree of calcification, and the distance from the sinuses [2]. In such a situation, some measures must be taken to prevent and treat CAO.

Prevention strategies such as coronary guidance, balloon, provisional stent, and catheter have been documented [3]. However, we still do not have systematization criteria for the selection of a device in certain situations.

We present a case with coronary obstruction during the procedure, which was successfully treated with chimney stent technique.

## Clinical case

**Fig. 1:**
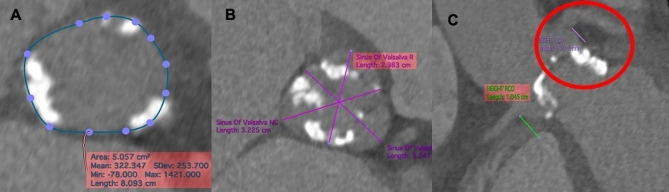
TAVR planning with Cardiac CT. **(A)** Aortic annulus with severe calcification, **(B)** Aortic valve with tricuspid morphology, **(C)** with severe calcification predominantly in the left sinus that extends to the LV outflow tract

An 84-year-old male with a history of high blood pressure and diabetes was admitted to our center for heart failure (NYHA III) and syncope. The echocardiogram reported aortic stenosis with a maximum gradient of 110 mmHg, a mean gradient of 62 mmHg, a valve area of ​​0.8 cm, an indexed area of ​​0.3 cm. /m2. Coronary angiography revealed a lesion in 70% of the proximal segment of the left anterior descending (LAD) artery. Due to high surgical risk, the Heart Team decided on TAVR and PCI to the LAD artery. During the planning of the tomography, a tricuspid valve with severe calcification was observed, the distance to the left ostium was 7 mm, distance from the right ostium of 10 mm, aortic annulus area 505 mm2, aortic annulus perimeter of 80.9 mm, sinus of valsalva right 29 mm, left 32 mm, and non-coronary 32 mm (Fig. 1).

### Step by step of the procedure

**Fig. 2:**
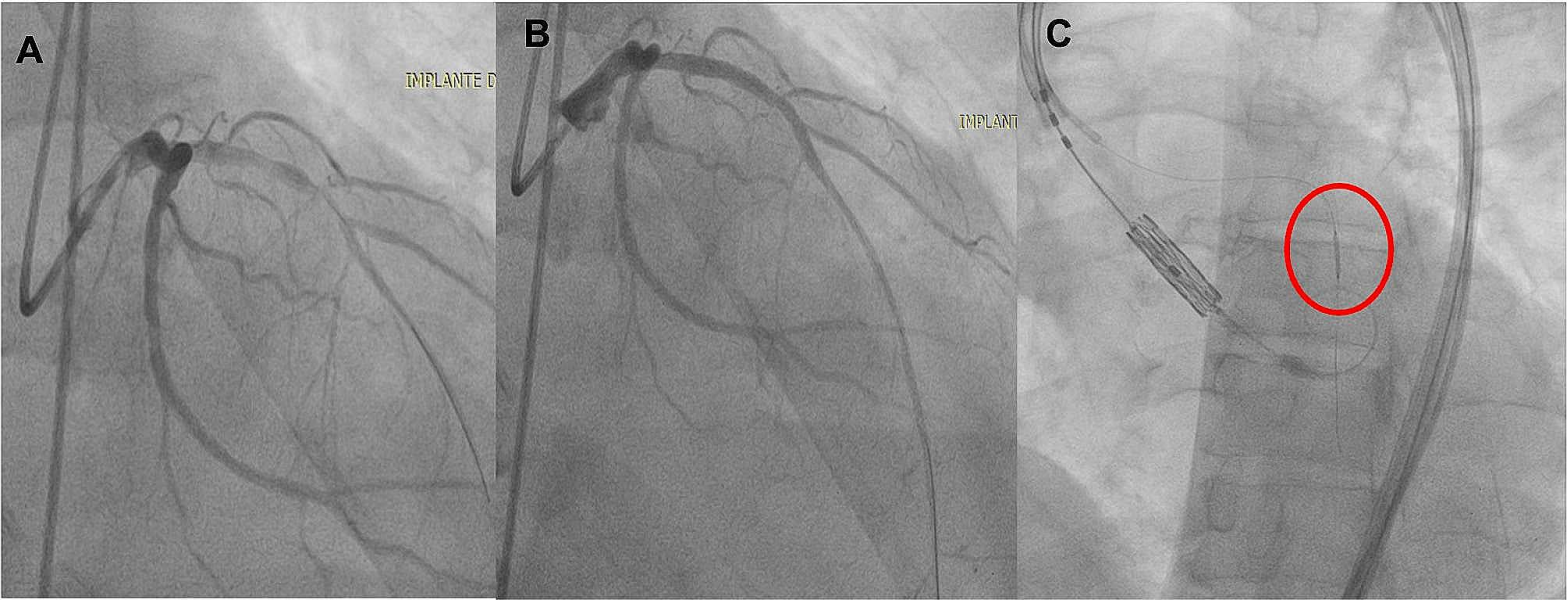
Procedural steps for coronary protection. **(A)** angiography showing a lesion in the middle segment of the anterior descending artery (blue arrow). **(B)** coronary intervention with placement of a 3.0 × 33 mm stent (red arrow). **(C)** Through the VODA catheter (brown arrow), advanced the stent distally with a diameter that was guided by IVUS

In the first step of the procedure through a left femoral approach and with a VODA catheter, percutaneous revascularization of the anterior descending artery was performed with placement of a 3.0 × 33 mm stent in the proximal segment. In the same procedure, IVUS measurement was performed on the LMCA, which reported a diameter of (5.2 mm) (Fig. 2) and, subsequently, a provisional stent (5.0 × 15 mm) was placed at the level of the proximal segment of the LAD artery (Fig. 2).

**Fig. 3:**
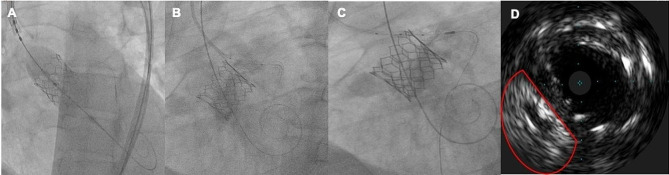
Procedural steps for chimney stenting. **A)** The guide catheter was removed away from the aortic valve, the transcatheter heart valve edwards #26 was deployed. **B and C)** Immediate decrease in blood flow of the left system was observed post-TAVR and the coronary stent is retracted and deployed within the proximal part of the coronary artery, extending above the displaced leaflet tissue and stent frame, Chimney stent under-expansion was observed due to calcium displacement observed with IVUS

In the second step of the procedure through the right femoral route, the release system was ascended, and we used the left radial access to ascend the pigtail catheter. Under conscious sedation and supported by pacing at 180 bpm, predilatation was performed with a #22 balloon and subsequently a #26 Edward Sapiens 3 aortic valve was placed (Fig. 3**and video 1 of supplemental material).** However, during valve release, he immediately presented hemodynamic instability and sustained ventricular tachycardia. Angiographically, there was a decrease in coronary flow in the left arterial system (Fig. 3). So, we performed retraction of the provisional stent until the aorta and valve frame with chimney technique **(video 2 of supplemental material**), (fourth step). We released the provisional stent and improvement in clinical status was observed instantly.

**Fig. 4:**
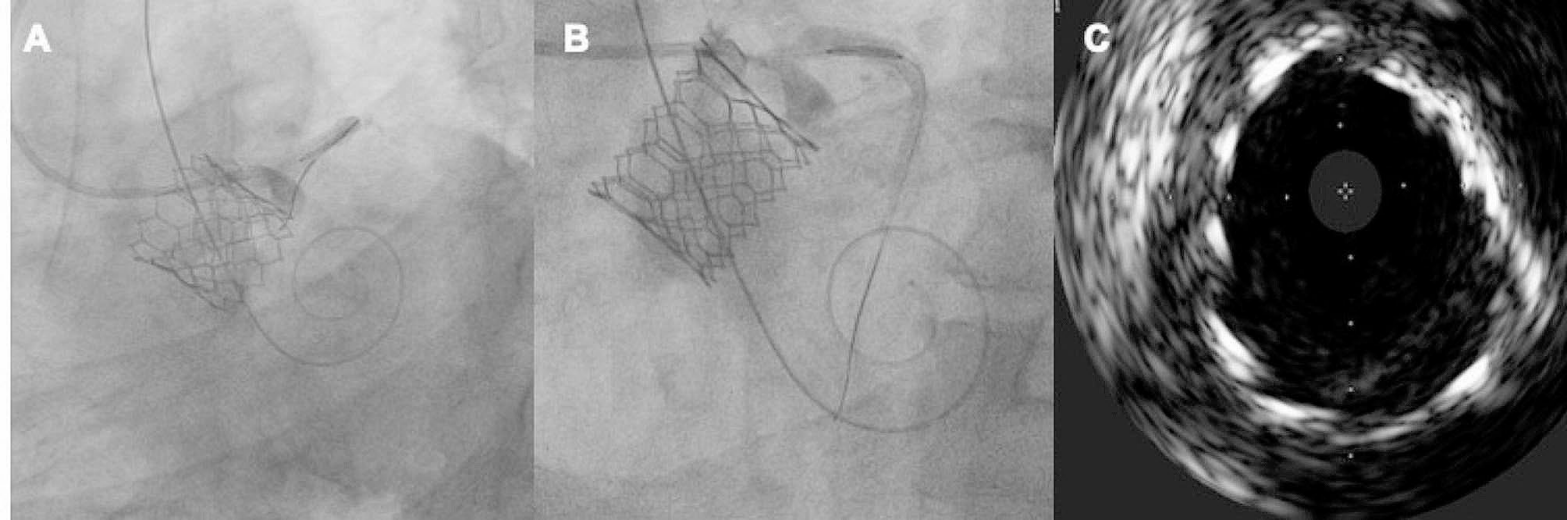
**A and B**) post-chimney stent-dilatation was performed. **C)** coronary stent with adequate expansion and minimum luminal area greater than 8 mm

In the fourth step, we performed a review with intravascular imaging where we observed underexpansion of the stent due to displacement of calcium material (Fig. 3). We performed postdilation of the stent with a 5.0 × 12 mm NC Trek balloon. The second review with IVUS showed an 8 mm luminal area, with TIMI III angiographic flow (Fig. 4**and video 3 of supplemental material)**. The aortography showed aortic valve without paravalvular leak (**video 4 of supplemental material)** The control echocardiogram reported a mean gradient of 8mmHg and velocity of 1.7 m/s. He was discharged 72 h after the procedure, and at 12-month follow-up he maintains functional class I.

**Fig. 5:**
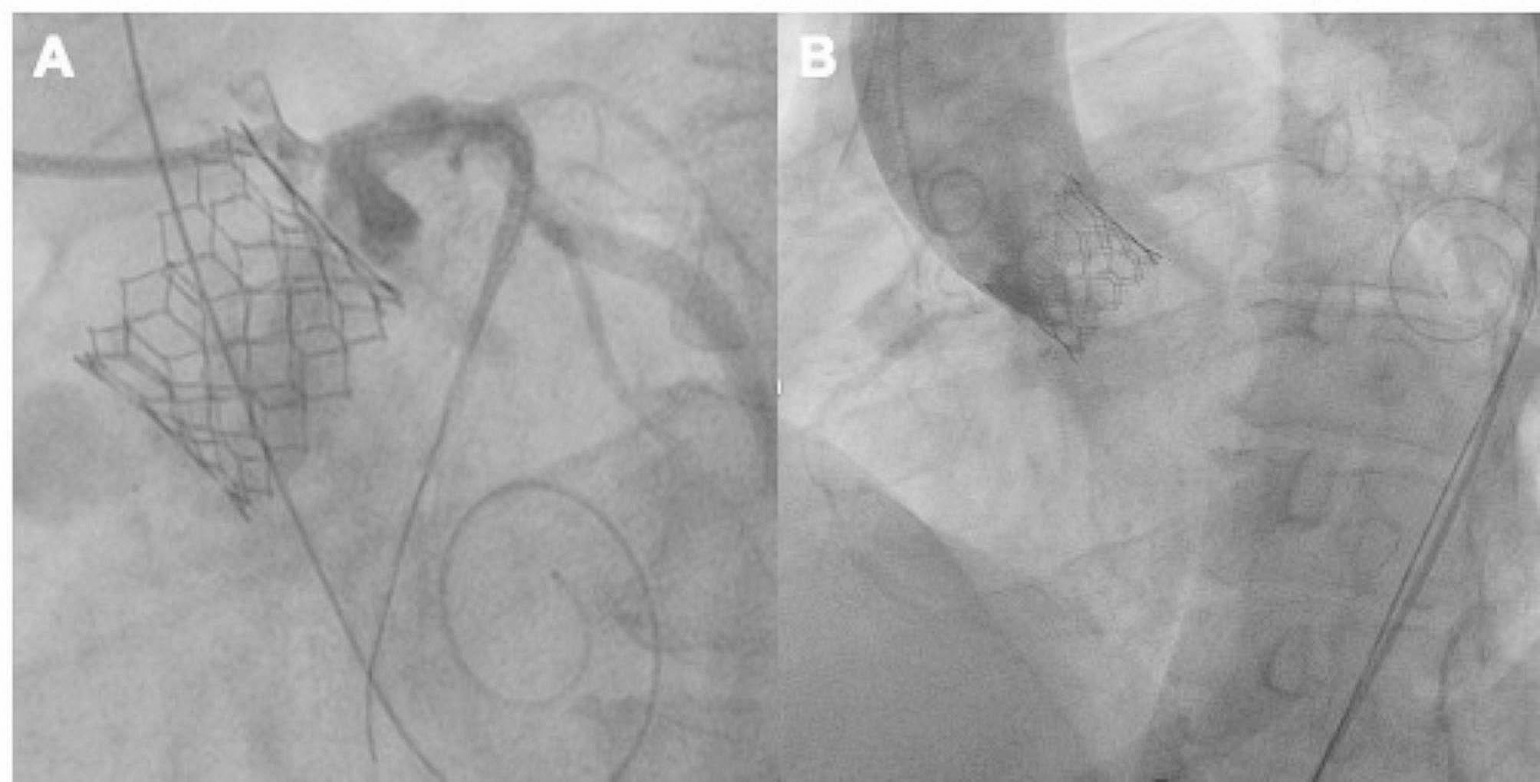
Control angiography **A**) improvement of the flow of the left system. **B**) aortography with aortic valve without paravalvular leak

## Discussion

Acute CAO is defined as a new complete or partial obstruction of one or both coronary ostia during a TAVI procedure [4]. This complication typically manifests as abrupt haemodynamic instability with rapid progression to cardiogenic shock and ventricular arrhythmias. The incidence of acute CAO during TAVR in native aortic valves is relatively low (< 1%) with a year mortality rate of 45.5%. In a large multicenter registry, the obstruction was most common on the left side (78.3%) [1, 5].

Pathophysiologically, the CAO occurs when the transcatheter heart valve (THV) displaces the underlying surgical or native aortic valve leaflets outward and obstructs the coronary artery ostia, directly or by sequestering the sinus of Valsalva at the sinotubular junction. However, CAO may be secondary to other less common mechanisms, such as the displacement of calcium towards the ostium.

Although the outcomes of patients undergoing TAVR are progressively improving because of the rapid evolution of technology, better imaging, increased operator experience, and the continuous iteration of devices, CAO remains a threatening complication with high rates of morbidity and mortality [6]. There are various predictors of CAO prior to valve placement such as the distance from the ostia, the degree of calcification, the distance from the sinuses. in such a situation, it highlights the importance of anticipating this complication. Therefore, some measures must be taken to prevent and treat coronary obstruction [2, 3].

Prevention strategies depend on the type of valve affected, whether it is native or bioprosthetic. Regarding bioprosthetic valves, the bioprosthetic aortic scallop intentional laceration to prevent iatrogenic coronary artery obstruction technique is a preventive measure (basilic technique). In native valve stenosis, there are currently 3 strategies to prevent coronary occlusion after TAVR: prophylactic guidewire, catheter, or stent intubation of at-risk coronary arteries.

In COPROTAVR registry, after a 3-year follow-up, the clinical outcome was generally favourable in patients treated with stenting. Preventive stent implantation across the coronary ostia was associated with good mid-term survival rates and low rates of stent thrombosis (cardiac mortality 7.8%, MI 9.8%, stroke 5.4%). In that trial, the patients protected with wires only had numerically more cardiac deaths compared to patients treated with stenting across the coronary ostia [7].

The rapidity of restoration of coronary blood flow appears to be an important determinant of outcome after CAO. Although a guide wire-only strategy its safe, it can be difficult to advance a coronary stent alongside the deployed THV (and displaced native leaflets) due to obstructing calcification or jailing of the safety wire between the aortic wall and the THV frame. A coronary balloon or stent premounted on the protective 0.014” guidewire can be parked distally in the coronary artery, retrieved proximally and deployed with rapid restoration of coronary flow in case of acute CAO [1, 5, 8]. However, stent jailing and difficulty in reaccessing coronary arteries must be taken into account as potential drawbacks of this technique.

Coronary access in this type of scenario is extremely challenging. The type of coronary revascularization in CAO depends on the anatomical characteristics of the aortic root and the perceived risk of CAO [8]. Two strategies have been implemented: the “chimney stent technique” coronary stenting across the coronary ostia with large protrusion in the aorta, and the “regular ostial stent technique” implying minimal protrusion of the stent in the aorta. In our case, we decided to perform the chimney technique due to the type of valve used and the anatomical characteristics of the patient [8–10].

The type of valve in patients with a high risk of obstruction has not yet been established. Due to the very complex anatomy and high risk of paravalvular leak, we decided to use an expandable balloon valve. One study suggested easier reaccess in Balloon expandible THV systems due to THV frame, and subsequently, opening the possibilty to do the chimney stent technique toward the aorta [10].

In our case, the high degree of calcification and the left ostium near the annulus were conditions for ostium obstruction at the time of valve release. In this context, provisional stenting prior to TAVR with a high risk of obstruction was an effective and safe strategy to achieve procedural success. It is therefore suggested that a protective coronary guide wire with a premounted coronary stent should be positioned distally in the coronary vessel prior to THV deployment.

**Video 1**: valve deployment.

**Video 2**: chimney technique.

**Video 3**: angiographic control of the left coronary arterial system.

**Video 4**: aortographic control.

### Electronic supplementary material

Below is the link to the electronic supplementary material.

Supplementary Material 1

Supplementary Material 2

Supplementary Material 3

Supplementary Material 4
